# The effect of long chain polyunsaturated fatty acid supplementation on intelligence in low birth weight infant during lactation: A meta-analysis

**DOI:** 10.1371/journal.pone.0195662

**Published:** 2018-04-10

**Authors:** Yuan Song, Ya Liu, Yun Pan, Xiaofeng Yuan, Pengyu Chang, Yuan Tian, Weiwei Cui, Dong Li

**Affiliations:** 1 Department of Nutrition and Food Hygiene, School of Public Health, Jilin University, Changchun, China; 2 Department of Gastroenterology, Jilin Province People’s Hospital, Changchun, China; 3 Tianqiao District of Ji’nan Food and Drug Administration, Ji’nan, Shandong Province, China; 4 Department of Pediatrics, Affiliated Hospital of Changchun University of Chinese Medicine, Changchun, China; 5 Department of Radiotherapy, The First Hospital of Jilin University, Changchun, China; 6 Key Laboratory for Molecular Enzymology and Engineering of the Ministry of Education, College of Life Sciences, Jilin University, Changchun, China; 7 Department of Immunology, College of Basic Medical Sciences, Jilin University, Changchun, China; 8 Department of Hepatology, The First Hospital of Jilin University, Changchun, China; Laval University, CANADA

## Abstract

**Background:**

Low birth weight infant (LBWIs) are prone to mental and behavioural problems. As an important constituent of the brain and retina, long chain polyunsaturated fatty acids are essential for foetal infant mental and visual development. The effect of lactation supplemented with long chain polyunsaturated fatty acids (LCPUFA) on the improvement of intelligence in low birth weight children requires further validation.

**Methods:**

In this study, a comprehensive search of multiple databases was performed to identify studies focused the association between intelligence and long chain polyunsaturated fatty acid supplementation in LBWIs. Studies that compared the Bayley Scales of Infant Development (BSID) or the Wechsler Abbreviated Scale of Intelligence for Children (WISC) scores between LBWIs who were supplemented and controls that were not supplemented with LCPUFA during lactation were selected for inclusion in the meta-analysis.

**Results:**

The main outcome was the mean difference in the mental development index (MDI) and psychomotor development index (PDI) of the BSID and the full scale intelligence quotient (FSIQ), verbal intelligence quotient (VIQ) and performance intelligence quotient (PIQ) of the WISC between LBWIs and controls. Our findings indicated that the mean BSID or WISC scores in LBWIs did not differ between the supplemented groups and controls.

**Conclusion:**

This meta-analysis does not reveal that LCPUFA supplementation has a significant impact on the level of intelligence in LBWIs.

## Introduction

Low birth weight infants (LBWIs) are born with a birth weight of less than 2500 g, and those with a birth weight less than 1500 g are referred to as very low birth weight infants (VLBWIs) [1]. Worldwide, LBWIs accounted for 15% to 20% of all newborns in 2014; most LBWIs were born in low- or middle-income countries: 28% of LBWIs were born in South Asia, approximately 13% in South Africa, and 9% in Latin America [2]. The mortality rate of 1500–1999 g weights LBWIs is approximately 2.8 times that of 2000–2499 g LBWIs and approximately 8 times that of normal weight newborns (>2500 g) [3]. Low birth weight (LBW) in infants has become a public health problem worldwide.

LBW is an important factor in the development of mental and psychomotor intelligence. Cognitive and physical development was reported to be negatively correlated with LBW [4], and LBW may result in cognitive dysfunction, mental retardation or cerebral palsy at early ages [5]. A previous meta-analysis showed that cognitive impairment associated with LBW will continue into adolescence and adulthood [6]. LBW and preterm birth often occur simultaneously, and because the most prolific period of foetal brain development is during the last three months of pregnancy [7], preterm birth can cause developmental immaturity of the neonatal nervous system.

The growth and development of LBWIs are closely related to their nutritional status. First, LBWIs require 110–150 calories daily, with additional milk supplementing that ingested from nursing in order to increase the carbohydrate intake [8]. Second, LBWIs require higher protein intake than normal newborns and need special formulas [9]. Since humans cannot synthesize n-3 and n-6 polyunsaturated fatty acids in vivo, they must get them from their diet, and LBWIs cannot effectively convert the precursor fatty acids, resulting in less capacity for fat storage [10]. LBWIs may lack long chain polyunsaturated fatty acids (LCPUFA) after birth, including docosahexaenoic acid (DHA) and arachidonic acid (AA). DHA and AA are essential for the development of the brain and central nervous system, and they quickly accumulate in the foetal anaphase and affect the development of the nervous system [11]. Previous reports studying whether supplementing breastfeeding with LCPUFA can improve LBWI intelligence were not conclusive. Some reports [12, 13] showed that supplementation with DHA and AA can improve infant intelligence. However, other studies [14, 15] showed that LBWIs supplemented with DHA and AA led to no significant improvement in neurodevelopment or in levels of intellectual, language, and motor development. The LCPUFA supplement dose, duration, ratio of different fatty acids, supplementation scheme and feeding patterns may impact the nervous system development and intelligence of LBWIs. Therefore, whether LCPUFA supplementation can improve neurodevelopment and intelligence, also, the duration and appropriate dose of LCPUFA supplementation require further investigation.

In this study, we conducted a meta-analysis to explore whether long chain polyunsaturated fatty acid supplementation can improve the intellectual level of LBWIs and to identify the most effective intervention duration.

## Materials and methods

### Sources and methods of data retrieval

We performed a comprehensive literature search that included studies until August 2017; the electronic databases searched included PubMed, Medline, Web of Science, and Google Scholar.

The searches were conducted to identify all published studies that reported data on the mean differences and standard deviations of the mental development index (MDI) and psychomotor development index (PDI) of the Bayley Scales of Infant Development (BSID) or the full scale intelligence quotient (FSIQ) of the Wechsler Abbreviated Scale of Intelligence for Children (WISC) between LBWIs supplemented with DHA and AA and those who were not. The following terms were used for the literature search: very low birth weight infants, low weight birth infants, premature infants, preterm infants, neonatal prematurity, long chain polyunsaturated fatty acids, fish oil, n-3 fatty acid, n-6 fatty acid, eicosapentaenoic acid, docosahexaenoic acid, arachidonic acid, and randomized controlled trial. The term ‘OR’ was used as the set operator to combine different sets of results. The MDI and PDI of the BSID and FSIQ, verbal intelligence quotient (VIQ) and performance intelligence quotient (PIQ) of the WISC were determined and used in a meta-analysis to understand how intelligence levels differ between LBWIs supplemented with LCPUFA those who were not. The test age of the subjects and the duration of supplementation, as well as other confounding factors, were also considered.

### Inclusion criteria

**Fig 1:**
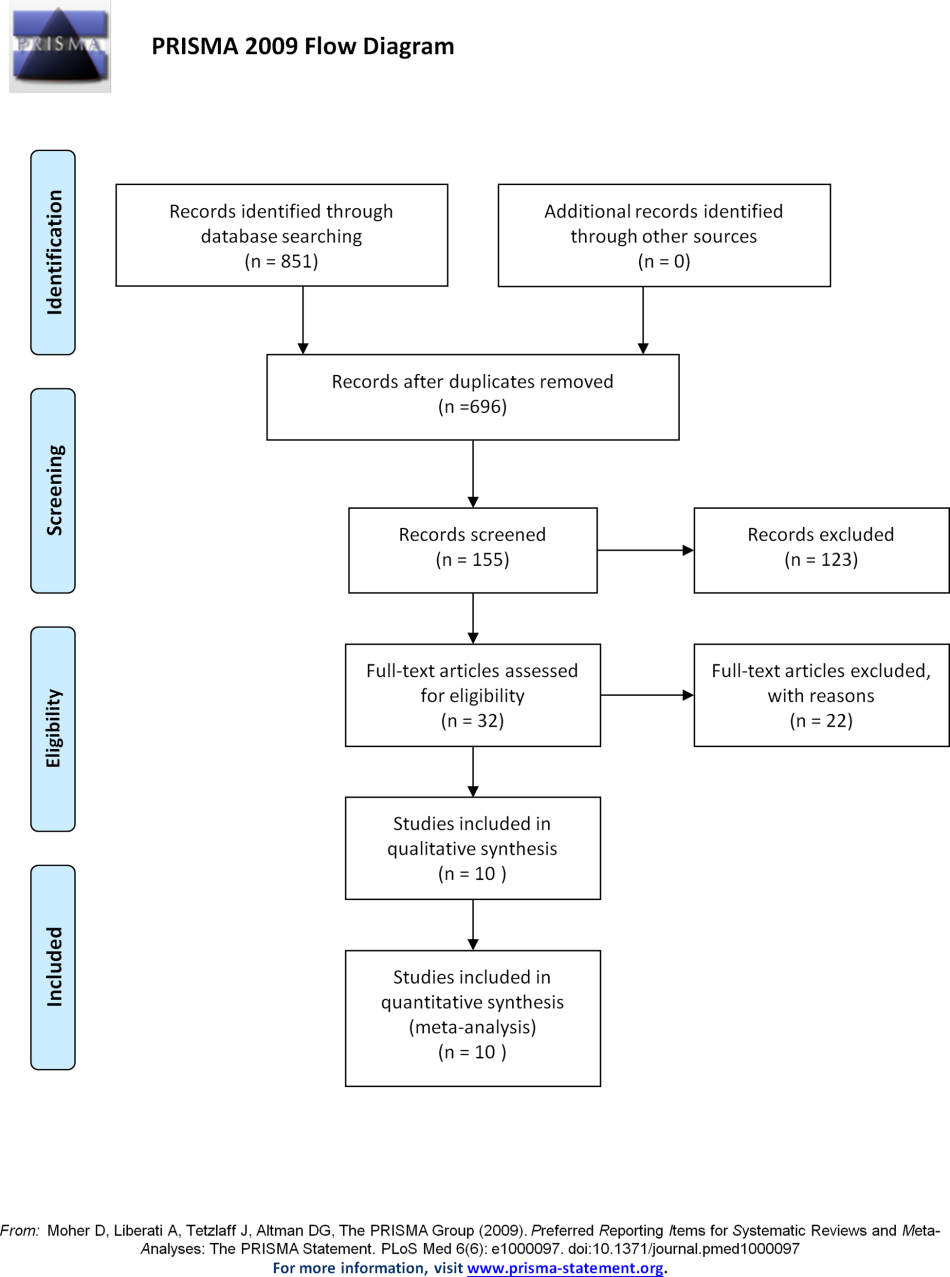
Flow chart of the selection of studies for the meta-analysis.

The articles that were included in this meta-analysis matched the following 7 criteria: (1) all study subjects were LBWIs with live birth weights of less than 2500 g; (2) the MDI and PDI of the BSID and the FSIQ, VIQ and PIQ of the WISC were presented as the mean ± standard deviation (SD); (3) LBWIs were randomly assigned to an intervention group that received long chain polyunsaturated fatty acid supplementation directly and a control group that did not; and (4) LBWIs were born without neonatal infection, disease that could have a significant effect on intelligence, or the intervention of LCPUFA could affect the treatment of any pre-existing diseases or conditions. (5) We made no restrictions on the feeding mode or supplied dose. (6) Guardians of subjects voluntarily signed the informed consent. (7) We excluded studies that did not provide initial data, studies in animals, in vitro studies, reviews and conference papers. Three investigators independently reviewed and extracted all the potentially eligible studies and discussed the inconsistencies until a consensus was reached (Fig 1). Additionally, the Jadad score was used to assess the quality of studies included in this meta-analysis [16].

### Data abstraction

We reviewed all the relevant studies and extracted the following data: (1) lead author, publication year, and sample size; (2) baseline data of the intervention group and the LBWI control group (sample size, sex, weight and gestational age); (3) feeding methods; and (4) the duration of the intervention, the intervention dose and the intelligence test ages (Tables 1 and 2).

**Table 1:** Details of studies employing the Bayley Scales of Infant Development.

Author	Year	Area	Sample(I/C)	Intervention time(mo)	Test age(mo)	Birth weight(I/C)	Gestational age(I/C)	Composition of long chain polyunsaturated fatty acids	MDI(I/C)	PDI(I/C)
Westerberg [13]	2010	Norway	40/42	2	20	1047(282) /1072(317)	28.6(2.9)/ 28.7(2.7)	DHA+AA	83.5±10.5/ 82.9±13.3	-
van Wezel-Meijler [14]	2002	Netherlands	22/20	6	3	1282(316)/ 1306(257)	30.4(1.5)/ 30.4(1.6)	DHA+AA	97.9±12.5/ 103.7±12.6	95.4±11.6/ 101.8±12.5
					6				98.5±17.7/ 106.8±10	97±13.5/ 105.7±10.2
					12				110.1±12.7/ 111.5±11.4	95.8±12.9/ 102.1±12.7
					24				101.9±18.4/ 97.7±21.5	101.5±15.5/ 105.1±12.6
O'Connor [17]	2001	USA, British and Chile	123/60	12	12	1305(293) /1287 (272)	29.8(2.1)/ 29.6(1.9)	DHA+AA	92.8±11.2/ 92.2±12.2*	87.2±14.2/ 86.3±16.2
			105/59			1309(286) /1287 (272)	29.7(2.0)/ 29.6(1.9)		93.4±13/ 92.2±12.2#	85.4±14.4/ 86.3±16.2
Woltil[18]	1999	Netherlands	13/37	1.5	19	2188(1085–2460) /2137(1120–2500)	37(31–41)/ 36(31–40)	DHA+EPA +DPA	101±9/ 103±15Δ	102±11/ 105±21
			13/37						107±12/ 103±15□	116±8/ 105±21
Fewtrell[24]	2004	British	112/116	9	18	1487(342)/ 1510(326)	31.1(1.9)/ 31.2(2.1)	DHA+AA +EPA	86.5±14.6/ 85.1±15.4	85.2±13/ 86.5±14.7
Fang[25]	2005	China	16/11	6	6	1980(110) /1990(120)	33.3(0.5) /33.0(0.5)	DHA+AA	96.1±8.6/ 91.7±10.4	102.2±10.5/ 95.4±13.2
				12					98.7±8/ 90.5±6.9	98±5.8/ 86.7±11.1
Fewtrell[26]	2002	British	78/80	1	12	1353(274)/ 1336(284)	30.3(2.4)/ 30.4(2.3)	DHA+AA +EPA	86.9±14.6/ 84.3±15	89.4±13.9/ 87.4±15

I: low birth weight with long chain polyunsaturated fatty acids supplementation. C: low birth weight without long chain polyunsaturated fatty acids supplementation. Mo: month.

* long chain polyunsaturated fatty acids from fish/fungal oil.

# long chain polyunsaturated fatty acids from egg-derived triglyceride/fish oil.

Δ supplementation with evening primrose oil (18:3ω6 0.32 mol/100 mol) and a single dosage of purified fish oil (LCPω-3 0.38mol/100 mol).

□ supplementation with evening primrose oil (18:3ω6 0.32 mol/100 mol) and a double dosage of purified fish oil (LCPω-3 0.8mol/100 mol).

MDI: mental development index of the Bayley Scales of Infant Development. PDI: psychomotor development index of the Bayley Scales of Infant Development. AA: Arachidonic acid. DHA: Docosahexaenoic acid. EPA: Eicosapentaenoic acid.

**Table 2:** Details of studies using the Wechsler Abbreviated Scale of Intelligence for Children.

Author	Year	Area	Sample(I/C)	Intervention time(mo)	Test age(year)	Birth weight(I/C)	Gestational age(I/C)	Composition of long chain polyunsaturated fatty acids	FSIQ(I/C)	VIQ(I/C)	PIQ(I/C)
Almaas[27]	2015	Norway	45/53	2	8.6	1028 (277)/ 1070 (315)	28.6(2.9)/ 28.6(2.6)	DHA+AA	92.7±8.8/ 93.9±10	88.8±10.3/ 90.3±12.5	95±12.6/ 95.9±14.4
Collins[28]	2015	Australia	291/313	9.3	7	1307(420)/ 1320(410)	30(28–31)/ 30(28–31)	DHA+AA	98.3±14/ 98.5±14.9	98±14.2/ 98.8±15.8	98.5±14.5/ 98.5±13.6
Isaacs[29]	2011	British	50/57	9	10	1454(369) 1512(338)	30.6(2.3)/ 30.9(2.0)	DHA+AA +EPA	95.1±13.2/ 92.7±12.3	96.7±13.2/ 92.6±12.6	94.2±12.7/ 94.5±14.1

I: low birth weight with long chain polyunsaturated fatty acid supplementation. C: low birth weight without long chain polyunsaturated fatty acid supplementation. Mo: month.

FSIQ: full scale intelligence quotient of the Wechsler Abbreviated Scale of Intelligence for Children. VIQ: verbal intelligence quotient of the Wechsler Abbreviated Scale of Intelligence for Children. PIQ: performance intelligence quotient of the Wechsler Abbreviated Scale of Intelligence for Children. AA: Arachidonic acid. DHA: Docosahexaenoic acid. EPA: Eicosapentaenoic acid.

### Statistical analysis

All statistical analyses were conducted using the statistical software Stata (version 12.0, StataCorp LLC, College Station, TX, USA). The mean difference and standard deviation of the intelligence scores of the LBWIs and control group were used for the meta-analysis. We combined the weighted mean difference (WMD) for studies that reported mean and standard deviation values for the intelligence score of the LBWI and control groups. An inverse variance weighted random-effects model or fixed-effects model was used to determine the WMD and 95% confidence intervals (CIs) and to measure the different intelligence scores of the LBWI and control groups, and the results were used to evaluate the differences in intelligence levels of the LBWIs and controls. In two of the included studies, two treatment groups were examined: one study investigated supplementation with two different doses of long chain polyunsaturated fatty acids, and the other investigated two different sources of long chain polyunsaturated fatty acids [17, 18]. To avoid double counting, the controls in each of these 2 studies were split approximately evenly into 2 control groups with the means and standard deviations left unchanged before the results were included in the meta-analysis [19, 20].

We used Cochran’s Q statistic and the *I*^*2*^ statistic to assess the statistical heterogeneity of the meta-analysis [21]. If the data were homogeneous (*P* >0.05), a fixed-effects model meta-analysis was performed; if the data were heterogeneous (*P* ≤0.05), a random-effects model meta-analysis was performed. Heterogeneity was considered significant at *P* <0.05 in the Q test, and the *I*^*2*^ value was used to evaluate the degree of heterogeneity. We defined low, medium and high heterogeneity at *I*^*2*^ values of 25%, 50%, and 75%, respectively [22]. Subgroup analyses were performed for the intelligence test age and the duration of supplementation. We used a funnel plot to detect publication bias concerning this meta-analysis, with the symmetry of the funnel plot used to determine whether publication bias occurred. Furthermore, a formal statistical assessment of the funnel plot asymmetry was performed using Egger’s regression asymmetry test [23].

## Result

### Study characteristics

Our search identified 851 related references; however, only 10 papers met our inclusion criteria. The 10 studies included 1793 individuals in total, with 908 cases in the intervention group and 885 controls [13, 14, 17, 18, 24–29]. Of the 10 studies, 7 studied intelligence test score via the BSID [13, 14, 17, 18, 24–26] and 3 studies intelligence test via WISC [27–29]. Seven studies reported the MDI of the BSID [13, 14, 17, 18, 24–26]; 6 studies reported the PDI of the BSID [14, 17, 18, 24–26]; 3 studies reported the FSIQ, VIQ and PIQ of the WISC [24–26]; Three studies had an intervention duration of less than 3 months [13, 18, 26], 2 studies had an intervention duration of between 4 and 6 months [14, 25], and 2 studies had an intervention duration of 7–12 months [17, 24]. Two studies reported 0–12 month-old children's intelligence [14, 25], 5 studies reported 13–24 month-old children's intelligence [13, 17, 18, 24, 26], and 3 studies reported intelligence in children older than 7–10 years [27–29]; Wezel-Meijler et al’s study tested infants' intelligence at 3 months, 6 months, 12 months and 24 months, and Fang et al's study tested infants' intelligence at 6 months and 12 months. The mean birth weights in 8 studies were less than 1500 g [13, 14, 17, 24, 26–29], those in 2 studies were more than 1500 g [18, 25], none of the included studies had infants with birth weight less than 750 g. Three studies reported mean gestational ages less than 30 weeks [13, 17, 27], and 6 studies’ mean gestational ages were more than 30 weeks [14, 24–26, 28, 29]; Woltil et al’s study reported the median gestational age. The supplement compositions of long chain polyunsaturated fatty acids included DHA and AA in 4 studies; DHA, AA and EPA in 2 studies; and DHA, EPA and DPA in 1 study. O'Connor et al’s studied two different sources of long chain polyunsaturated fatty acids as a supplement: one from fish/fungal oil and the other from egg-derived triglyceride/fish oil. Woltil et al’s study used different doses of long chain polyunsaturated fatty acids, and Isaacs et al’s research used two different feeding methods—infant formula and breastfeeding supplementation—for the long chain polyunsaturated fatty acids and studied the effects on children's intelligence. The 10 studies included 3 studies conducted in Britain [17, 24, 26], 2 in Norway [13, 27], 2 in Netherlands [14, 18], 1 in Australia [28], 1 in China [25], and 1 study involving the United States, Britain and Chile [17]. The patients’ basic characteristics are presented in Tables 1 and 2.

### Bayley Scales of Infant Development

#### MDI

**Fig 2:**
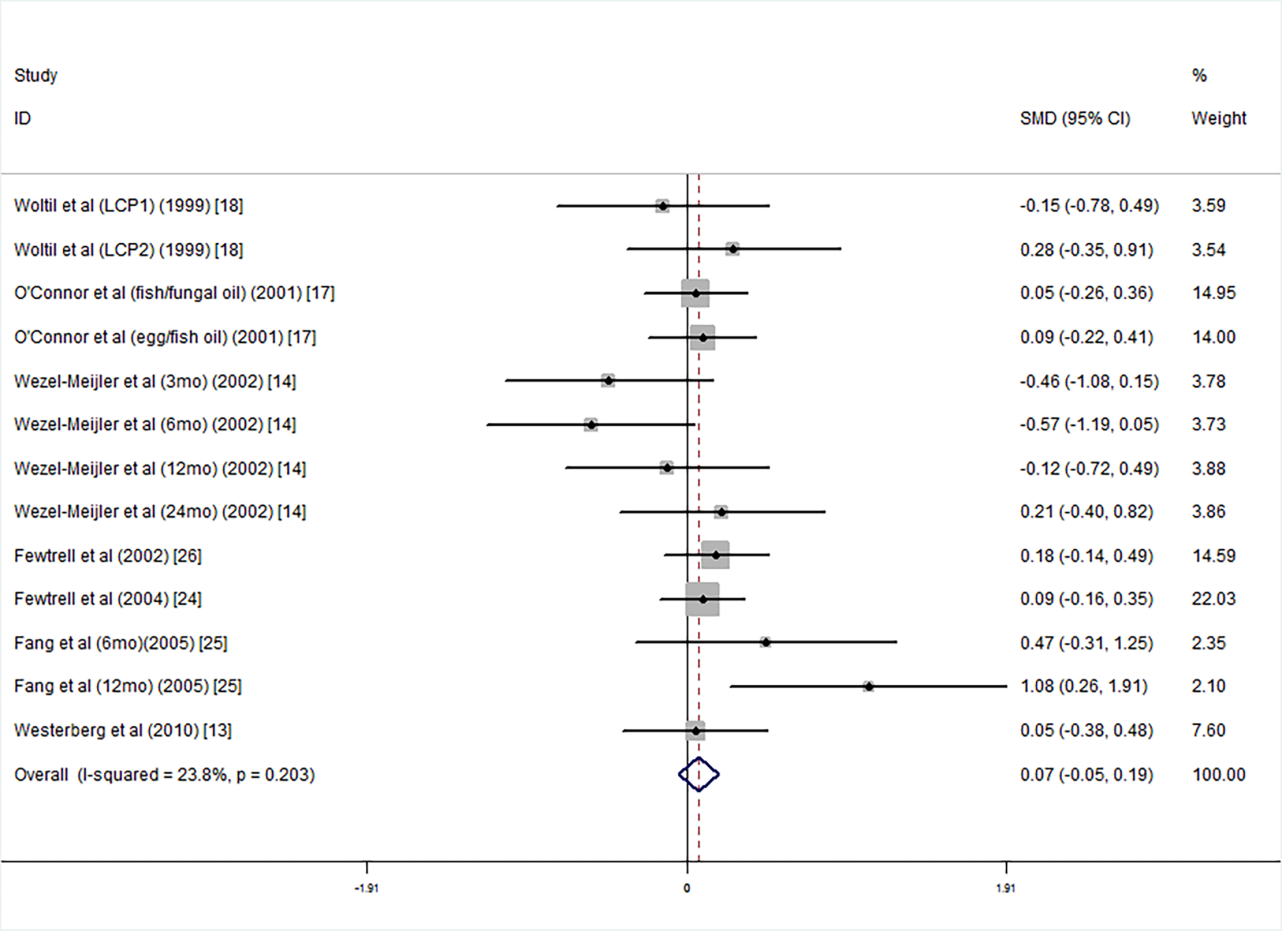
Forest plot of MDI scores in the long chain polyunsaturated fatty acid supplementation groups vs. controls; standardized mean differences with 95% confidence intervals and weight percentage are shown. Fish/fungal oil: long chain polyunsaturated fatty acids from fish/fungal oil. Egg/fish oil: long chain polyunsaturated fatty acids from egg-derived triglyceride/fish oil. LCP1: supplements of evening primrose oil (18:3ω6 0.32 mol/100 mol) and a single dose of purified fish oil (LCPω-3 0.38 mol/100 mol). LCP2: supplements of evening primrose oil (18:3ω6 0.32 mol/100 mol) and a double dose of purified fish oil (LCPω-3 0.8 mol/100 mol).

For studies that used the BSID to test infants’ intelligence before 24 months of age, the results show that the long chain polyunsaturated fatty acids supplemented group did not have significantly higher MDI scores than the group without supplementation (SMD = 0.07, 95% CI = −0.05, 0.19, *I*^*2*^ = 23.8%, P = 0.222; Fig 2). The study heterogeneity value was *I*^*2*^ = 23.8%, P = 0.203, showing that the heterogeneity of the studies was small; publication bias results showed that there was no evidence of publication bias (Egger's test: coefficient = 0.036, P = 0.968).

**Fig 3:**
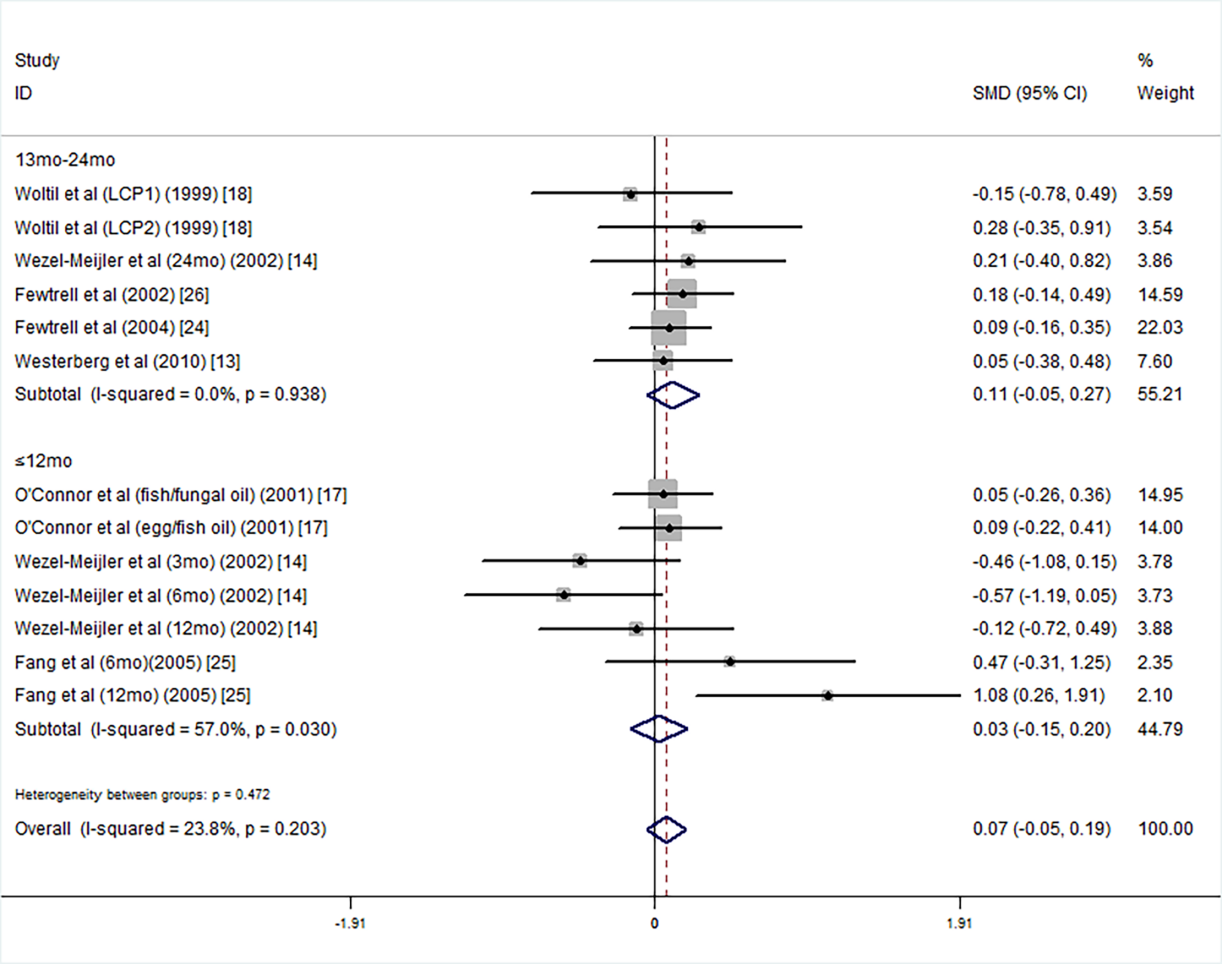
Forest plot of the MDI scores of long chain polyunsaturated fatty acid supplementation groups vs. controls by testing age groups; standardized mean differences with the 95% confidence interval and weight percentage are shown. Subtotals are for testing ages less than 12 mo and for ages of 13 mo-24 mo. Fish/fungal oil: long chain polyunsaturated fatty acids from fish/fungal oil. Egg/fish oil: long chain polyunsaturated fatty acids from egg-derived triglyceride/fish oil. LCP1: supplements of evening primrose oil (18:3ω6 0.32 mol/100 mol) and a single dose of purified fish oil (LCPω-3 0.38 mol/100 mol). LCP2: supplements of evening primrose oil (18:3ω6 0.32 mol/100 mol) and a double dose of purified fish oil (LCPω-3 0.8 mol/100 mol).

We conducted subgroup analyses on the intelligence testing age, supplementation duration, mean birth weight, and gestational age. For the intelligence testing age, we divided the data into two subgroups of 0 mo-12 mo old [14, 25] and 13 mo-24 mo old [13, 14, 17, 18, 24–26]. Because Wezel-Meijler's study tested infants' intelligence at 3 months, 6 months, 12 months and 24 months, the findings were divided into a 0 mo-12 mo old group and a 13 mo-24 mo old group. Subgroup analysis showed that the LCPUFA supplementation groups did not have significantly higher MDI scores than the control groups (Fig 3).

**Fig 4:**
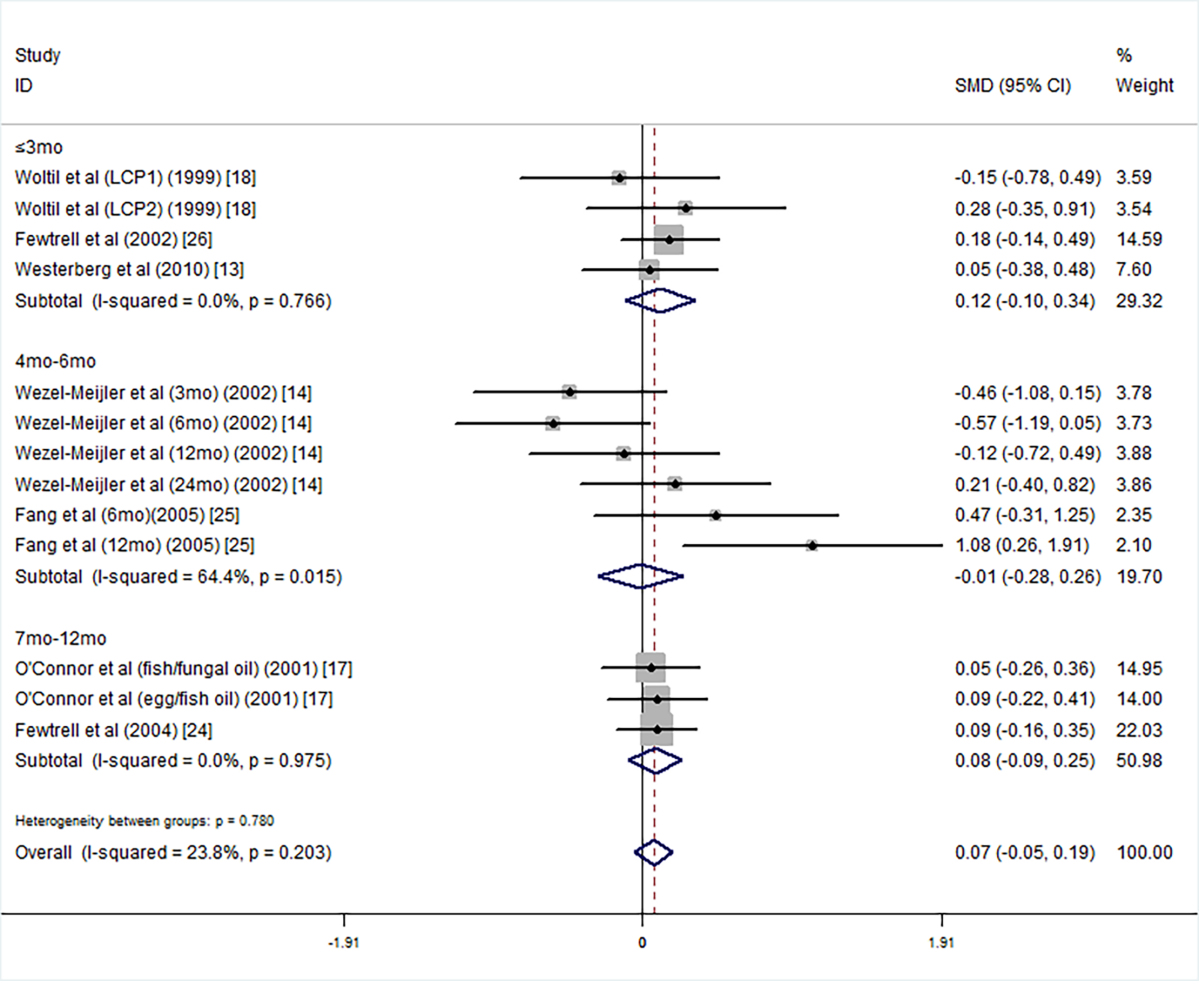
Forest plot of the MDI scores by supplementation duration for the long chain polyunsaturated fatty acid supplementation groups vs. controls; standardized mean differences with the 95% confidence interval and weight percentage are shown. Subtotals are for the forest plot of the MDI scores for supplementation durations of less than 3 mo, 4 to 6 mo, and 7–12 mo. Fish/fungal oil: long chain polyunsaturated fatty acids from fish/fungal oil. Egg/fish oil: long chain polyunsaturated fatty acids from egg-derived triglyceride/fish oil. LCP1: supplements of evening primrose oil (18:3ω6 0.32 mol/100 mol) and a single dose of purified fish oil (LCPω-3 0.38 mol/100 mol). LCP2: supplements of evening primrose oil (18:3ω6 0.32 mol/100 mol) and a double dose of purified fish oil (LCPω-3 0.8 mol/100 mol).

Next, we carried out a subgroup analysis according to the supplementary duration. The results shown are for supplementary duration less than 3 months [13, 18, 26], 4 months to 6 months [14, 25], or more than 7 months [17, 24], for this analysis, the MDI scores of the intervention groups were not higher than those of the control groups (Fig 4).

**Fig 5:**
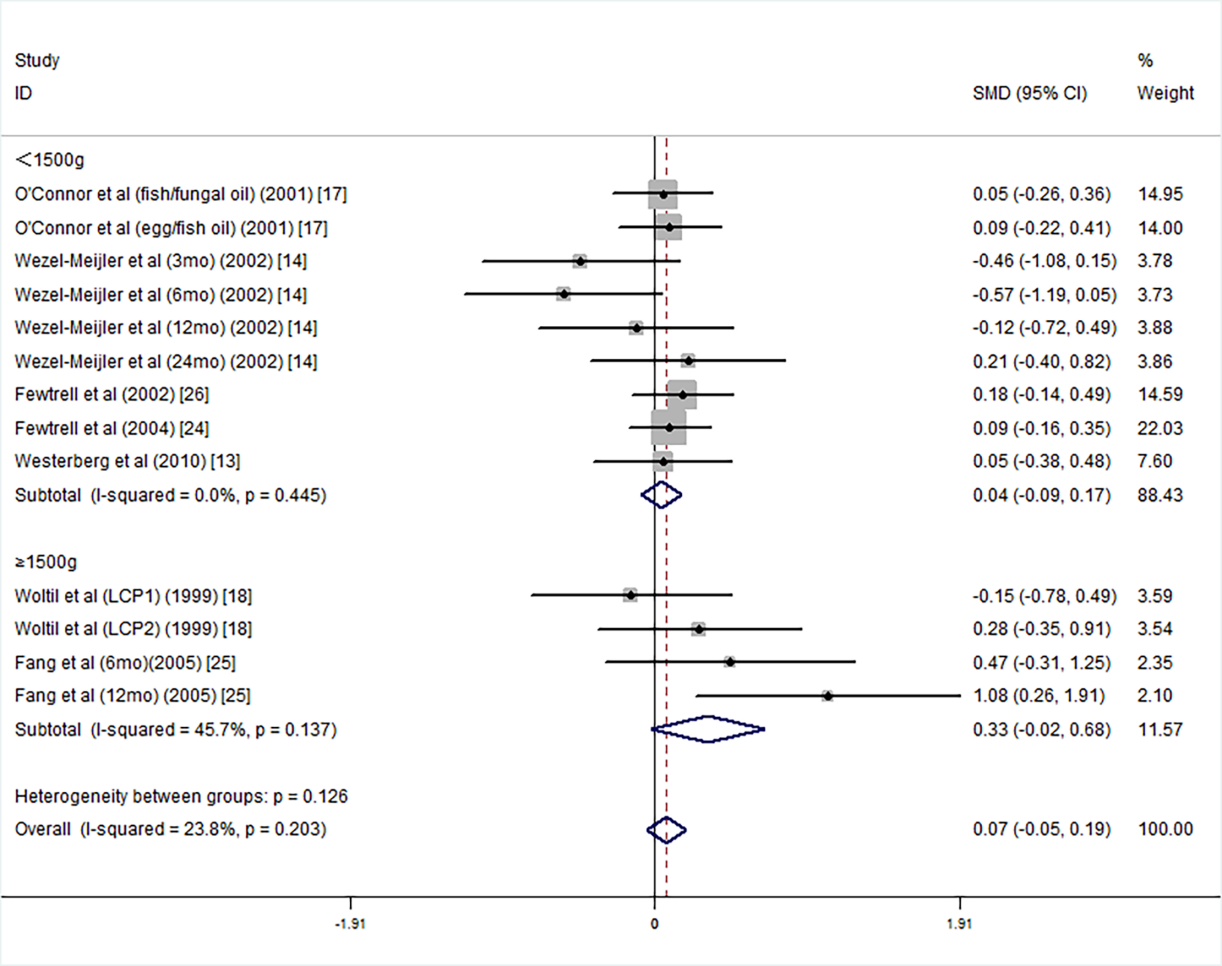
Forest plot of the MDI score in the different mean birth weight of long chain polyunsaturated fatty acids supplementation groups vs. controls; standardized mean differences with the 95% confidence interval and weight percentage are shown. Subtotals are for the Forest plot of the MDI score in the different birth weight at less than 1500g and more than 1500g. Fish/fungal oil: long chain polyunsaturated fatty acids from fish/fungal oil. Egg/fish oil: long chain polyunsaturated fatty acids from egg-de-rived triglyceride/fish oil. LCP1: supplements of evening primrose oil (18:3ω6 0.32 mol/100 mol) and a single dosage of purified fish oil (LCPω-3 0.38mol/100 mol). LCP2: supplements of evening primrose oil (18:3ω6 0.32 mol/100 mol) and double dosage of purified fish oil (LCPω-3 0.8mol/100 mol).

We divided the 7 studies into two subgroups of VLBWI [13, 14, 17, 24, 26–29] and LBWI [18, 25] according to the birth weight. The studies in the VLBWI and LBWI subgroups did not show differences in MDI scores between the LCPUFA-supplemented group and the controls (Fig 5).

**Fig 6:**
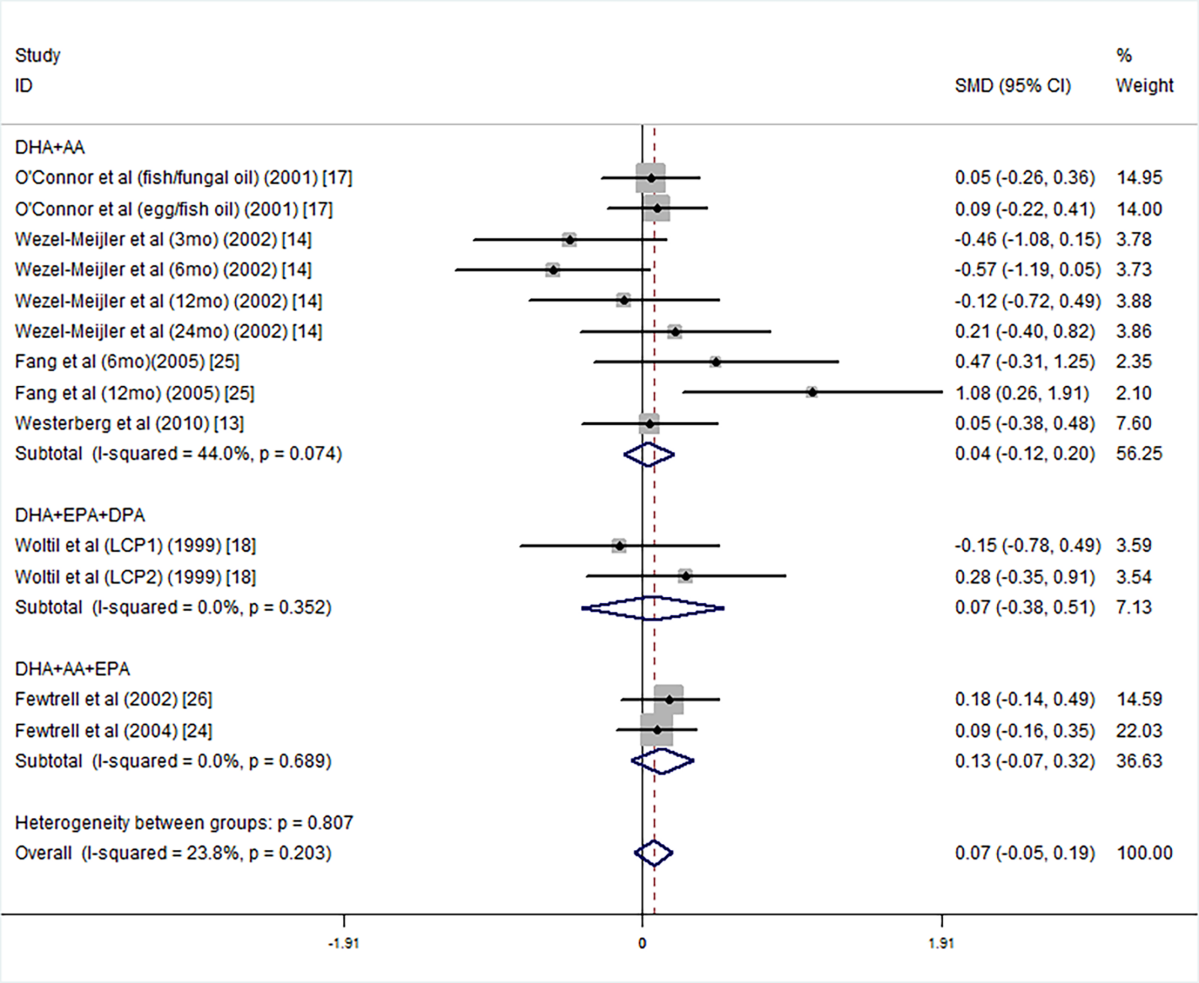
Forest plot of the MDI scores for different components of long chain polyunsaturated fatty acid supplementation groups vs. controls; standardized mean differences with the 95% confidence interval and weight percentage are shown. Subtotals are for the forest plot of the MDI scores with supplement compositions of DHA+AA, DHA+EPA+DPA and DHA+AA+EPA. Fish/fungal oil: long chain polyunsaturated fatty acids from fish/fungal oil. Egg/fish oil: long chain polyunsaturated fatty acids from egg-de-rived triglyceride/fish oil. LCP1: supplements of evening primrose oil (18:3ω6 0.32 mol/100 mol) and a single dose of purified fish oil (LCPω-3 0.38 mol/100 mol). LCP2: supplements of evening primrose oil (18:3ω6 0.32 mol/100 mol) and a double dose of purified fish oil (LCPω-3 0.8 mol/100 mol).

We carried out further subgroup analysis according to the components of fatty acids, DHA+AA [13, 14, 17, 25], DHA+EPA+DPA [18] or DHA+AA+EPA [24, 26]. statistically significant differences in MDI scores were not observed between the long chain polyunsaturated fatty acids supplied group and the controls (Fig 6).

#### PDI

**Fig 7:**
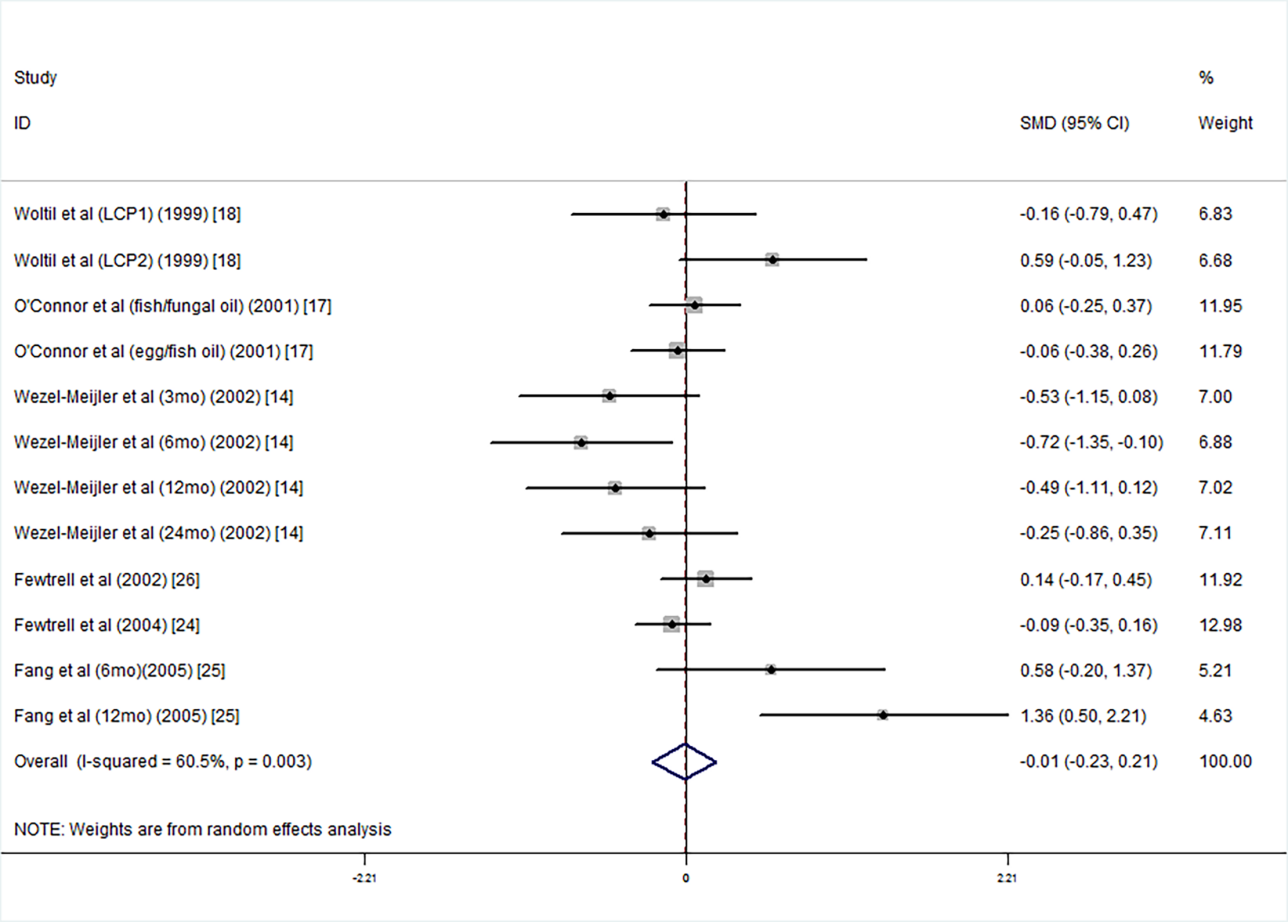
Forest plot of PDI scores in the long chain polyunsaturated fatty acid supplementation groups vs. controls; standardized mean differences with the 95% confidence interval and weight percentage are shown. Fish/fungal oil: long chain polyunsaturated fatty acids from fish/fungal oil. Egg/fish oil: long chain polyunsaturated fatty acids from egg-derived triglyceride/fish oil. LCP1: supplements of evening primrose oil (18:3ω6 0.32 mol/100 mol) and a single dose of purified fish oil (LCPω-3 0.38 mol/100 mol). LCP2: supplements of evening primrose oil (18:3ω6 0.32 mol/100 mol) and a double dose of purified fish oil (LCPω-3 0.38 mol/100 mol).

The results of these studies indicate that the LCPUFA supplementation group did not have significantly higher PDI scores than the group without supplementation (SMD = −0.01, 95% CI = −0.23, 0.21, *I*^*2*^ = 60.5%, P = 0.906; Fig 7). The studies’ heterogeneity statistics were as follows: *I*^*2*^ = 60.5%, P = 0.003, and from these data, we consider the studies to show significant heterogeneity; publication bias results showed that there was no evidence of publication bias (Egger's test: coefficient = 0.42, t = 0.35, P = 0.737).

**Fig 8:**
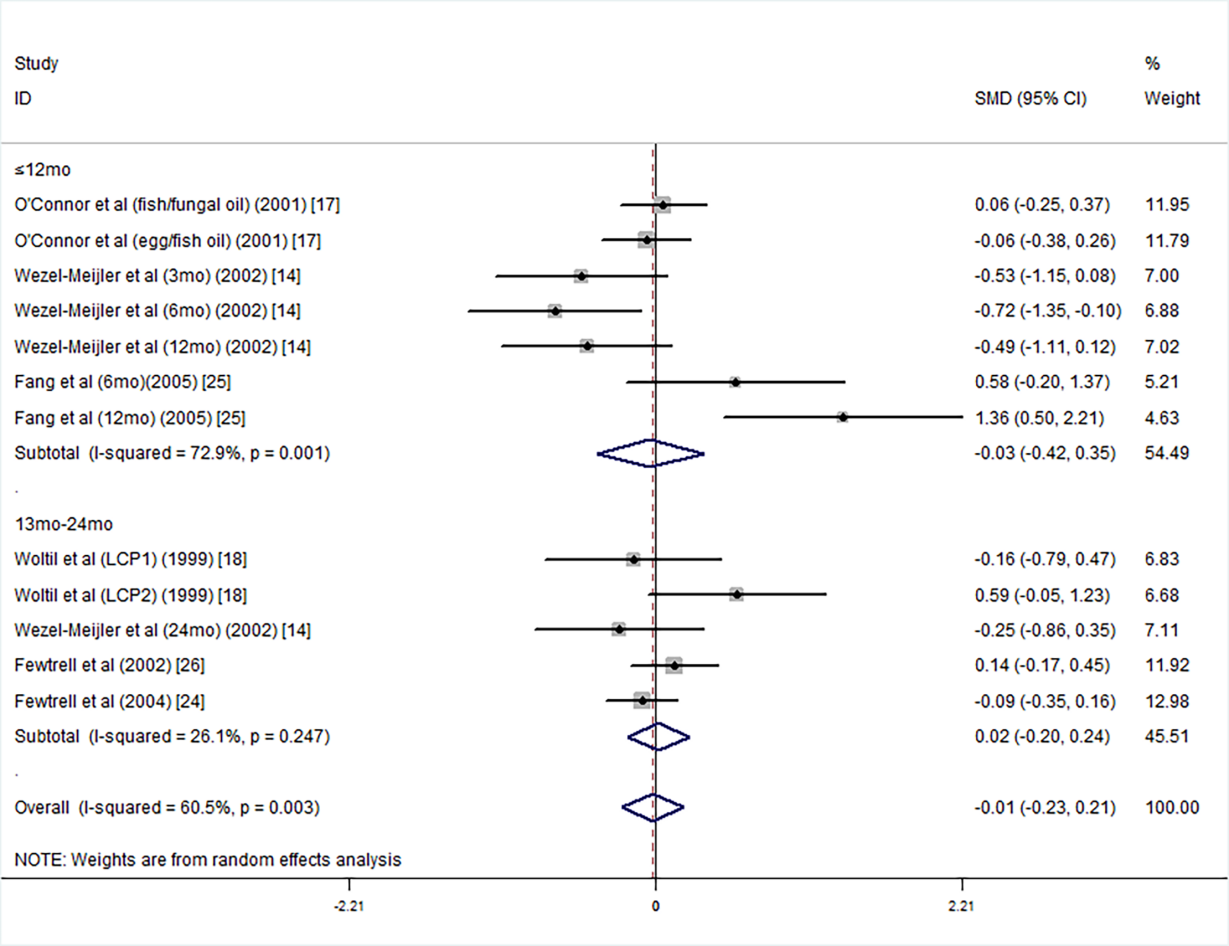
Forest plot of the PDI scores by testing age for the long chain polyunsaturated fatty acid supplementation groups vs. the controls; standardized mean differences with the 95% confidence interval and weight percentage are shown. Subtotals are for the testing age less than 12 mo and 13 mo-24 mo. Fish/fungal oil: long chain polyunsaturated fatty acids from fish/fungal oil. Egg/fish oil: long chain polyunsaturated fatty acids from egg-derived triglyceride/fish oil. LCP1: supplements of evening primrose oil (18:3ω6 0.32 mol/100 mol) and a single dose of purified fish oil (LCPω-3 0.38 mol/100 mol). LCP2: supplements of evening primrose oil (18:3ω6 0.32 mol/100 mol) and a double dose of purified fish oil (LCPω-3 0.8 mol/100 mol).

We conducted subgroup analyses according to the intelligence testing age, supplementation duration, mean birth weight, and gestational age. According to the intelligence testing age, we divided into the subjects into two subgroups of 0 mo-12 mo old [14, 25] and 13 mo-24 mo old [13, 14, 17, 18, 24–26]. Because Wezel-Meijler’s study tested infants' intelligence at 3 months, 6 months, 12 months and 24 months, the results were divided into a 0 mo-12 mo-old group and a 13 mo-24 mo-old group. Subgroup analysis showed that the LCPUFA supplementation groups did not have significantly higher PDI scores than the control groups (Fig 8).

**Fig 9:**
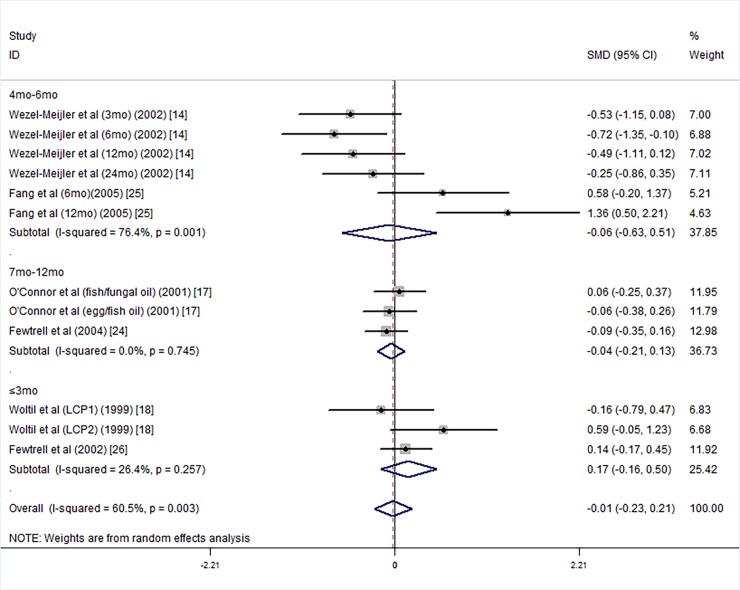
Forest plot of the PDI scores in groups with different durations of supplementation with long chain polyunsaturated fatty acids vs. the controls; standardized mean differences with the 95% confidence interval and weight percentage are shown. Subtotals are for the forest plot of the PDI scores for the supplementation durations of less than 3 mo, 4 to 6 mo, and 7–12 mo. Fish/fungal oil: long chain polyunsaturated fatty acids from fish/fungal oil. Egg/fish oil: long chain polyunsaturated fatty acids from egg-de-rived triglyceride/fish oil. LCP1: supplements of evening primrose oil (18:3ω6 0.32 mol/100 mol) and a single dose of purified fish oil (LCPω-3 0.38 mol/100 mol). LCP2: supplements of evening primrose oil (18:3ω6 0.32 mol/100 mol) and a double dose of purified fish oil (LCPω-3 0.8 mol/100 mol).

Next, we carried out subgroup analyses according to supplementary duration. The results were for supplementary durations less than 3 months [13, 18, 26], 4 months to 6 months [14, 25], or more than 7 months [17, 24]; the PDI scores of the intervention groups were not higher than those of the control groups (Fig 9).

**Fig 10:**
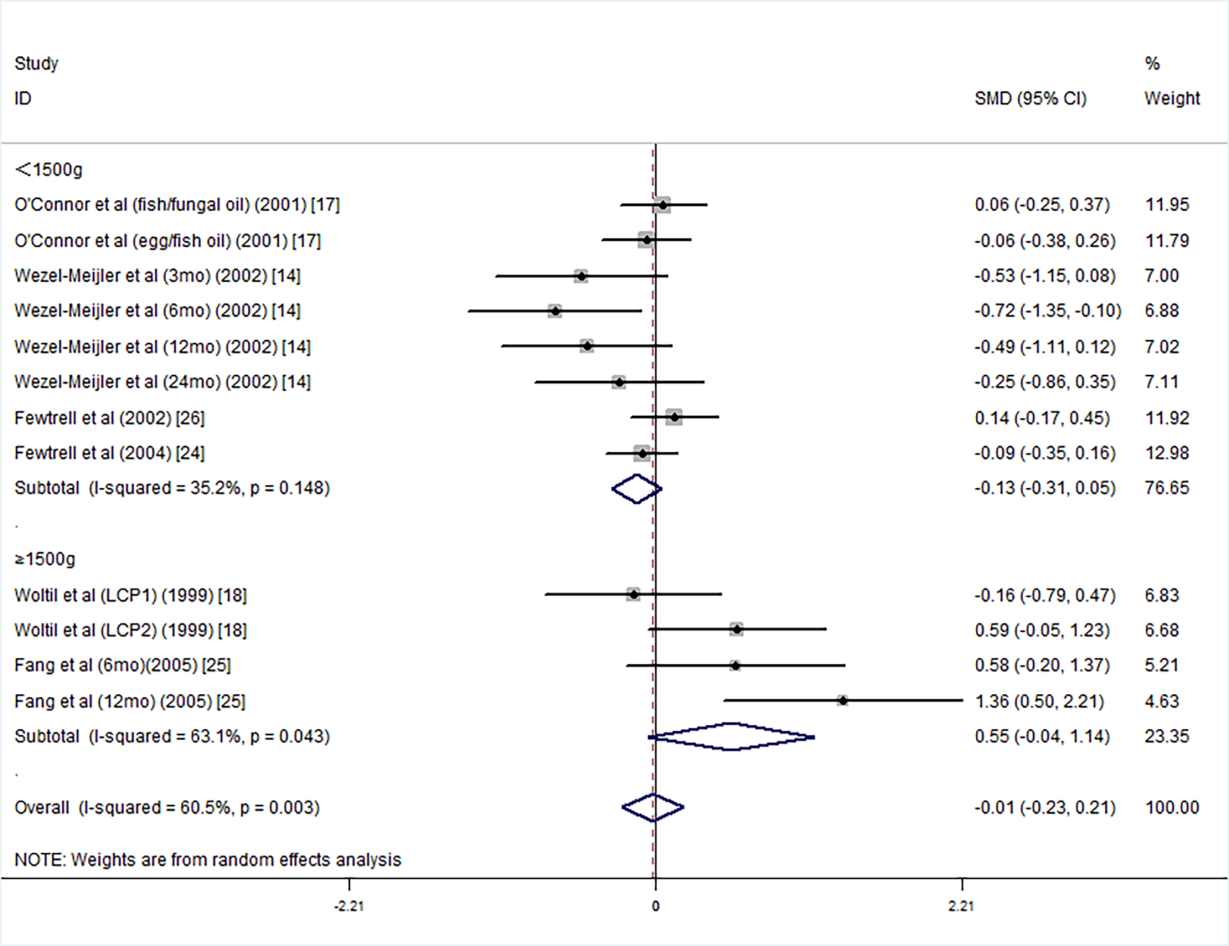
Forest plot of the PDI scores by mean birth weight for the long chain polyunsaturated fatty acid supplementation groups vs. the controls; standardized mean differences with the 95% confidence interval and weight percentage are shown. Subtotals are for the forest plot of the PDI scores in the supplementation duration groups of less than 1500 g and more than 1500 g. Fish/fungal oil: long chain polyunsaturated fatty acids from fish/fungal oil. Egg/fish oil: long chain polyunsaturated fatty acids from egg-derived triglyceride/fish oil. LCP1: supplements of evening primrose oil (18:3ω6 0.32 mol/100 mol) and a single dose of purified fish oil (LCPω-3 0.38 mol/100 mol). LCP2: supplements of evening primrose oil (18:3ω6 0.32 mol/100 mol) and a double dose of purified fish oil (LCPω-3 0.8 mol/100 mol).

We divided the 7 studies into two subgroups of VLBWI [13, 14, 17, 24, 26–29] and LBWI [18, 25] according to the mean birth weight. The studies in subgroups of VLBWI and LBWI did not show differences in the PDI scores between the LCPUFA group and the controls (Fig 10).

**Fig 11:**
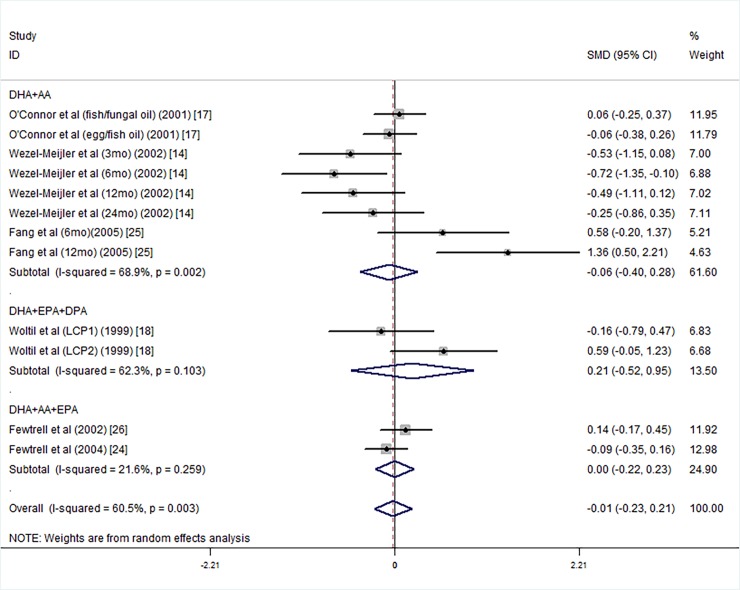
Forest plot of the PDI scores by the different components of polyunsaturated fatty acid supplementation groups vs. the controls; standardized mean differences with the 95% confidence interval and weight percentage are shown. Subtotals are for the forest plot of the MDI scores for the different supplement compositions of DHA+AA, DHA+EPA+DPA and DHA+AA+EPA. Fish/fungal oil: long chain polyunsaturated fatty acids from fish/fungal oil. Egg/fish oil: long chain polyunsaturated fatty acids from egg-derived triglyceride/fish oil. LCP1: supplements of evening primrose oil (18:3ω6 0.32 mol/100 mol) and a single dose of purified fish oil (LCPω-3 0.38 mol/100 mol). LCP2: supplements of evening primrose oil (18:3ω6 0.32 mol/100 mol) and a double dose of purified fish oil (LCPω-3 0.8 mol/100 mol).

We carried out further subgroup analysis according to the components of fatty acids, DHA+AA [13, 17, 25], DHA+EPA+DPA [18] or DHA+AA+EPA [24, 26]. statistically significant differences in PDI scores were not observed between the LCPUFA supplied group and the controls (Fig 11).

### Wechsler Abbreviated Scale of Intelligence for Children (WISC)

**Fig 12:**
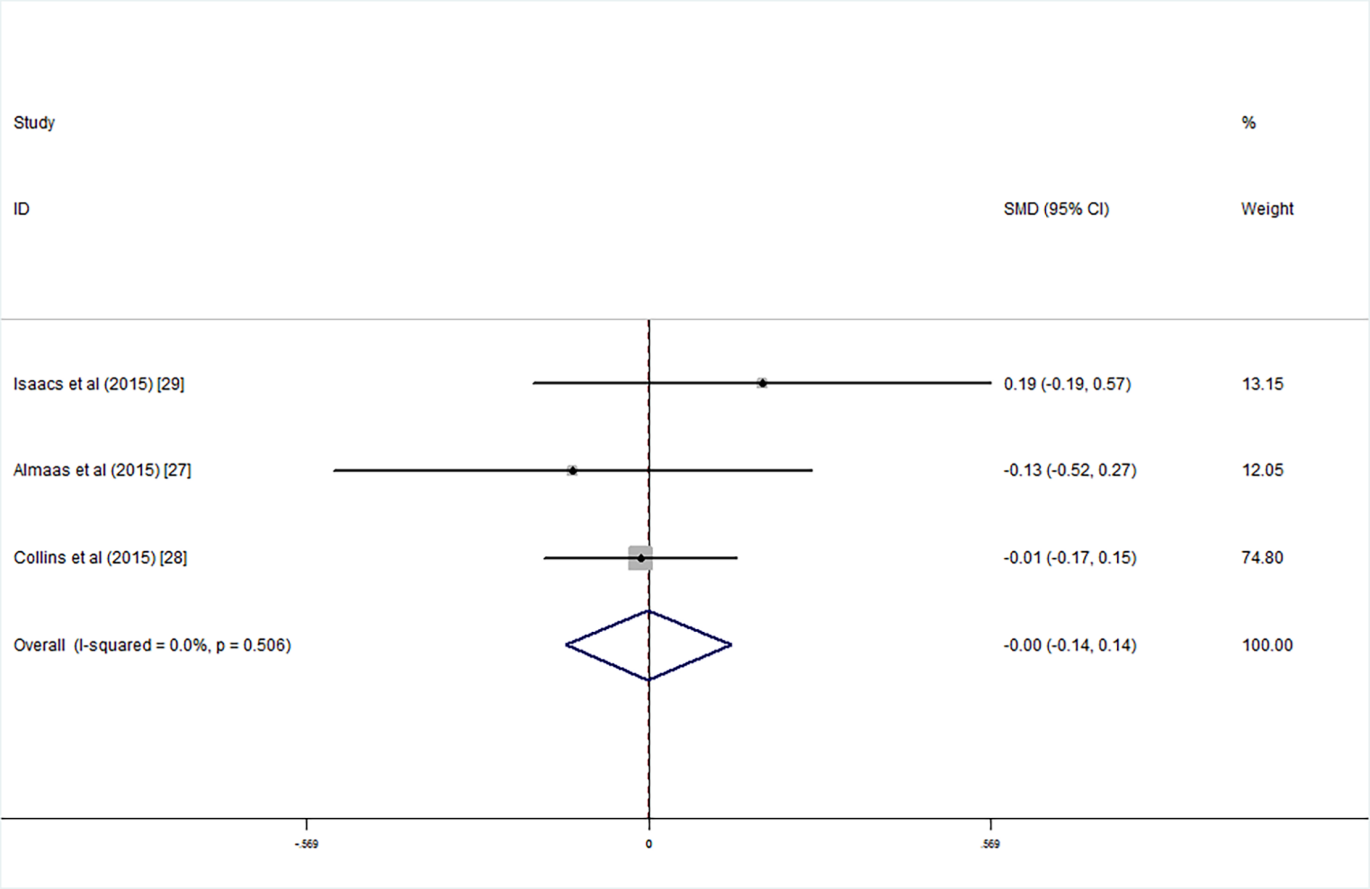
Forest plot of FSIQ score in the long chain polyunsaturated fatty acid supplementation groups vs. the controls; standardized mean differences with the 95% confidence interval and weight percentage are shown.

**Fig 13:**
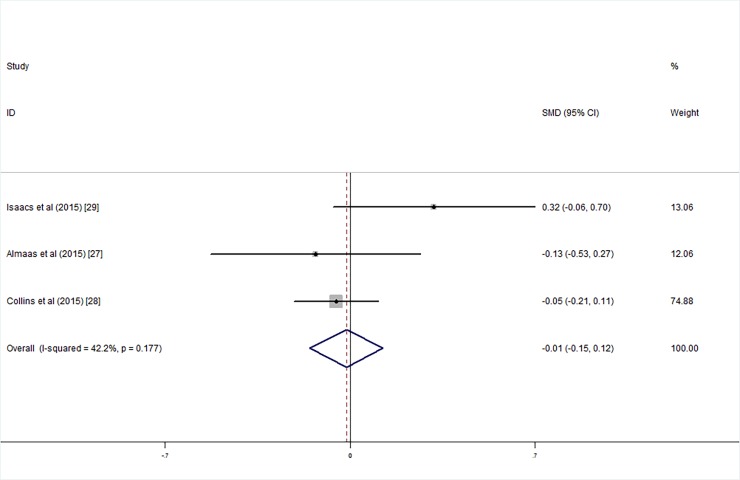
Forest plot of VIQ score in the long chain polyunsaturated fatty acids supplementation groups vs. the controls; standardized mean differences with the 95% confidence interval and weight percentage are shown.

**Fig 14:**
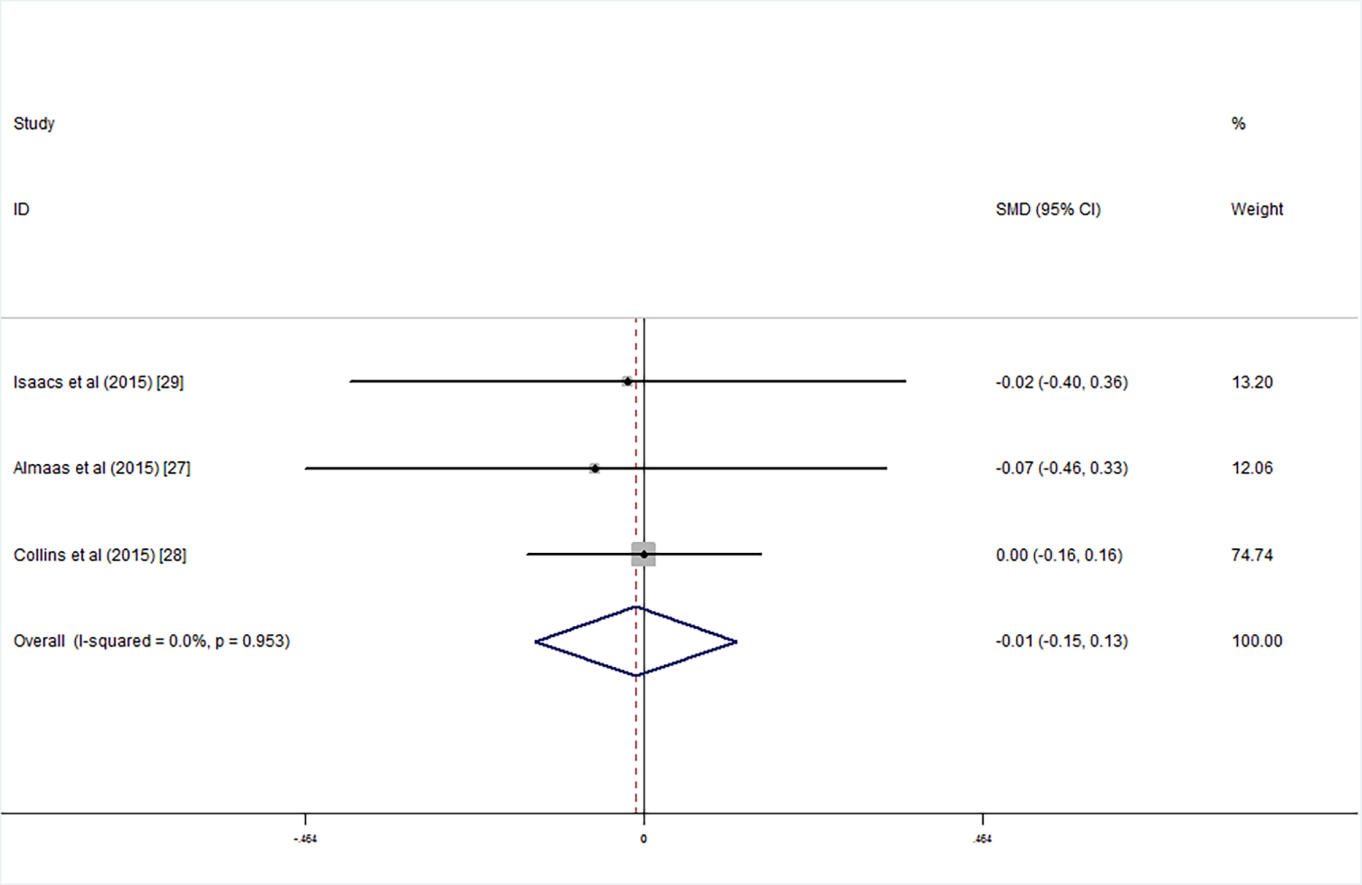
Forest plot of PIQ score in the long chain polyunsaturated fatty acid supplementation groups vs. the controls; standardized mean differences with the 95% confidence interval and weight percentage are shown.

Three studies employed the WISC with low birth weight intelligence tested at an average age of 7–10 years; meta-analysis results showed that there were no significant differences between the children who were supplemented with LCPUFA after birth and the control group with respect to the FSIQ (SMD_FSIQ_ = 0.00, 95% CI = -0.14, 0.14, *I*^*2*^ = 0.00%, P = 0.991; Fig 12), VIQ (SMD_VIQ_ = 0.01, 95% CI = -0.15, 0.12, *I*^*2*^ = 42.2%, P = 0.844; Fig 13) and PIQ (SMD_PIQ_ = -0.01, 95% CI = -0.15, 0.13, *I*^*2*^ = 0.00, P = 0.877; Fig 14) scores. The studies’ heterogeneity was not significant (*I*^*2*^ = 0.00%, P = 0.506), and there was no publication bias (Egger's test: coefficient = 0.37, t = 0.24, P = 0.851).

## Discussion

Currently, LCPUFA supplements such as DHA or AA are widely used in infant formula, and this is become one of the "selling points" for a lot of brands at least in China, despite there is no conclusive answer to whether these supplements are required or indeed beneficial to infants [30]. Infant formula in China is already a very complicated issue [31, 32]; whether LCPUFA supplements had to be added in the infant formula further enhanced the complexity of this issue. Based on the results from this meta-analysis, LCPUFA supplements had no beneficial effect, at least on the intelligence development of low birth infants.

Although previous studies have shown that maternal intake of n-3 polyunsaturated fatty acids during pregnancy and lactation may promote the development of the infant’s nervous system and intelligence [12, 33–36], this meta-analysis could not support this hypothesis. Our analysis showed that there was no statistically significant difference in intelligence scores of LBWIs between LCPUFA supplemented groups and control groups. Subgroup analysis of the duration of interventions or the combinations of LCPUFA (DHA+AA, DHA+EPA+DPA or DHA+AA+EPA) had no effect on the intellectual level of the intervention group compare to that of the control group either.

Among ten studies we included in this meta-analysis [13, 14, 17, 18, 24–29], only 3 studies [18, 24, 25] fund LCPUFA supplement did help in mental development. And these three studies used PDI or MDI scores which were tested before 2 years of age, but developmental assessment of VLBWIs before 2 years of age might be not reliable for prediction of future mental development since prematurity might affect the test results [37]. In all the long-term follow up studies [27–29] that used the WISC scores which were tested at around 7–10 years of age, no beneficial effects of LCPUFA supplements in mental development were found. Taking together, we believe the hypothesis that LCPUFA supplement is required for LBWIs is highly debatable.

There are some limitations in this study. For example, among the studies that include different supplemental doses of LCPUFA, we could not calculate the relationship between the fatty acid supplement dose and infant intelligence. Therefore, future studies would be benefit from using different doses of DHA and AA or a different fatty acid ratio as a supplement for LBWIs, thereby exploring the appropriate LCPUFA supplemental doses and allocation ratios. The other issue we need to point out is that we excluded all the studies with LBWIs with neonatal infection or disease in this meta-analysis, as LBWIs are at high risk of pulmonary diseases [38] or other chronic disorders [39], whether LCPUFA supplements are indeed beneficial for these infants could not be answered by this analysis. Furthermore, there were some weaknesses in clinical studies of LCPUFA supplements: relatively short duration of intervention, variance in populations and the limitations of testing methods. So the beneficial effects of LCPUFA which were shown in animal studies might not be revealed in clinical studies due to these shortages. Ultimately, a multi-centre, randomized quality-controlled set of experiments with a large sample size is still needed to prove that LCPUFA supplements improve LBWI intelligence and to provide more reliable evidence.

## Conclusions

In summary, although long-chain polyunsaturated fatty acids were reported to be essential for foetal infant mental and visual development, the impact of DHA, AA, EPA or DPA supplementation on level of intelligence of LBWIs could not be proven. Therefore, whether long-chain polyunsaturated fatty acids supplements are beneficial for LBWIs has not been shown conclusively.

## Supporting information

S1 Checklist PRISMA 2009 checklist. (DOC)

## Abbreviations

AA: Arachidonic acid
BSID: Bayley Scales of Infant Development
CI: Confidence intervals
DHA: Docosahexaenoic acid
EPA: Eicosapentaenoic acid
FSIQ: full scale intelligence quotient
LBW: Low birth weight
LBWI: Low birth weight infant
LCPUFA: long chain polyunsaturated fatty acids
MDI: mental development index
PDI: psychomotor development index
PIQ: performance intelligence quotient
SMD: Standardized mean difference
VIQ: verbal intelligence quotient
VLBWI: very low birth weight infants
WISC: Wechsler Abbreviated Scale of Intelligence for Children
WMD: weighted mean difference
